# The origin of HTLV-1 in the South Bahia by phylogenetic, mitochondrial DNA and β-globin analysis

**DOI:** 10.1186/1742-4690-11-S1-P49

**Published:** 2014-01-07

**Authors:** Sandra R Gadelha, Milena M Aleluia, Marco AG Mello, Filipe FA Rego, Lucas S Pereira, Bernardo Galvão-Castro, Marilda Gonçalves, Sandra MB Sousa, Luiz C Alcântara

**Affiliations:** 1 Universidade Estadual de Santa Cruz, Ilhéus, Bahia, Brazil; 2 Fundação Osvaldo Cruz, Salvador, Bahia, Brazil; 3 Escola Bahia de Medicina e Saúde Pública/Fundação Bahiana para Desenvolvimento das Ciências, Salvador, Bahia, Brazil; 4 Universidade do Sudoeste da Bahia, Vitória da Conquista, Bahia, Brazil

## 

In order to clarify the HTLV introduction and dispersion in the south of Bahia, we analyzed 29 samples from HTLV-1 seropositive women. Before the blood collection, all of them answered a standardized questionnaire. The DNA was extracted by QIAgen Kit. It was performed a nested-PCR to the LTR region of the HTLV. The sequences were submitted to the LASP HTLV-1 Automated Subtyping Tool. The phylogenetic analyses were generated using the neighbor-joining e máxima-verossimilhança methods. The evolutionary model of Tamura-Nei with gamma distribution was selected as the best adaptive model by software Modeltest 3.7. The likelihood ratio was used to calculate the statistical support for the branches in trees. Bayesian tree were constructed to verify the posterior probability statistical parameter. To evaluate genetic ancestry of the population, it was analyzed mtDNA ancestry markers and β-globina haplotypes. From the total, 21 samples were successfully amplified and sequenced and they were classified as HTLV-1 aA (bootstrap support of 100%). The phylogeny analysis showed multiple introductions of the virus in Brazil. In addition, for the first time, Mozambique sequences were grouped with Brazilian and South Africa sequences, supporting the hypothesis that Africans infected with the virus have been brought from the southern regions of Africa. In relation to the genetic ancestry, the African ethnicity was predominantly found by mtDNA markers. In addition, the type benin was detected by β-globin analyses. These data corroborate to clarify the introduction and dispersion of this virus in Brazil, especially in the Bahia.

